# *APC* loss induces Warburg effect via increased *PKM2* transcription in colorectal cancer

**DOI:** 10.1038/s41416-020-01118-7

**Published:** 2020-10-19

**Authors:** Pu-Hyeon Cha, Jeong-Ha Hwang, Dong-Kyu Kwak, Eunjin Koh, Kyung-Sup Kim, Kang-Yell Choi

**Affiliations:** 1 Department of Biotechnology, College of Life Science and Biotechnology, Yonsei University, Seoul, Korea; 2 Department of Biochemistry and Molecular Biology, Integrated Genomic Research Center for Metabolic Regulation, Institute of Genetic Science, College of Medicine, Yonsei University, Seoul, Korea; 3 CK Biotechnology Inc., Building 117, 50 Yonsei Ro, Seodaemun-Gu, Seoul, Korea

**Keywords:** Colorectal cancer, Cell signalling, Cancer metabolism

## Abstract

**Background:**

Most cancer cells employ the Warburg effect to support anabolic growth and tumorigenesis. Here, we discovered a key link between Warburg effect and aberrantly activated Wnt/β-catenin signalling, especially by pathologically significant *APC* loss, in CRC.

**Methods:**

Proteomic analyses were performed to evaluate the global effects of KYA1797K, Wnt/β-catenin signalling inhibitor, on cellular proteins in CRC. The effects of *APC*-loss or Wnt ligand on the identified enzymes, PKM2 and LDHA, as well as Warburg effects were investigated. A linkage between activation of Wnt/β-catenin signalling and cancer metabolism was analysed in tumour of *Apc*^*min/+*^ mice and CRC patients. The roles of PKM2 in cancer metabolism, which depends on Wnt/β-catenin signalling, were assessed in xenograft-tumours.

**Results:**

By proteomic analysis, PKM2 and LDHA were identified as key molecules regulated by Wnt/β-catenin signalling. *APC*-loss caused the increased expression of metabolic genes including *PKM2* and *LDHA*, and increased glucose consumption and lactate secretion. Pathological significance of this linkage was indicated by increased expression of glycolytic genes with Wnt target genes in tumour of *Apc*^*min/+*^ mice and CRC patients. Warburg effect and growth of xenografted tumours-induced by *APC-*mutated-CRC cells were suppressed by PKM2-depletion.

**Conclusions:**

The β-catenin-PKM2 regulatory axis induced by *APC* loss activates the Warburg effect in CRC.

## Background

Cancer cells, in contrast with normal cells, exhibit an altered metabolism, increased aerobic glycolysis, and decreased oxidative phosphorylation.^1,2^ Elevated glucose uptake and lactate production regardless of oxygen availability, which is referred to as aerobic glycolysis or the Warburg effect, are a dominant phenotype of most cancer cells.^3^ This rewired metabolism is required for growth, proliferation, and survival of cancer cells and consequently promotes the initiation and progression of tumours.^4^ Recently, growing evidence has shown that the reprogramming of cancer metabolism is directly regulated by activation of oncogenes or loss of function mutations of tumour suppressors such as phosphoinositide 3-kinase (PI3K), hypoxia inducible factor-1 (HIF-1) and p53.^1,5–7^ In addition, many studies showed that mutations and abnormal levels of metabolic enzymes and altered amounts of several metabolites directly affect tumorigenesis in various types of human cancers.^8–11^ These results renew interest in cancer metabolism and suggest the rewired cancer metabolism as a potential therapeutic target for cancer therapy. However, the mechanistic basis by which cancer metabolism is controlled by oncogenes and tumour suppressor genes varies in different tumour types, and in most cases, the molecular mechanisms that induce the alterations in the expression of the metabolic enzymes are poorly understood. Furthermore, although cellular metabolism reprogramming is considered as a critical event during tumorigenesis, the regulatory mechanisms and signalling pathways that initiate and control it remain elusive.

The Wnt/β-catenin signalling pathway is a major pathway that regulates important biological processes including normal development and oncogenesis.^12^ Aberrant activation of Wnt/β-catenin signalling has been frequently found in various cancers. Colorectal cancer (CRC) is the most notable cancer type associated with activation of Wnt/β-catenin signalling because loss of function mutations of *adenomatous polyposis coli* (*APC*) occur in up to 90% of human CRC patients.^13^ APC plays a role as a gatekeeper in colorectal tumorigenesis, and *APC* loss initiates the pathogenesis via stabilisation and subsequent nuclear translocation of β-catenin for transcriptional activation of target genes involving cell proliferation and transformation.^13^ CRC development caused by this aberrant Wnt/β-catenin signalling is regarded to most likely result from the inappropriate activation of genes.^14^ Recently, speculative links between Wnt/β-catenin signalling and cancer metabolism have been poised in several cancers.^15–18^ However, regulation of cancer metabolism by Wnt/β-catenin signalling, especially *APC* mutations, implying the major pathological significance of activation of Wnt/β-catenin signalling and initiation of CRC, is poorly characterised.

In this study, through systematic proteomic analysis, we identified significant decreases in the levels of glycolytic enzymes such as pyruvate kinase M2 (PKM2) and lactate dehydrogenase A (LDHA) in DLD1 cells treated with KYA1797K, a small molecule that inhibits Wnt/β-catenin signalling by degradation of β-catenin.^19^ The Wnt/β-catenin-signalling-dependent regulation of the metabolic enzyme expression was confirmed by various in vitro studies. The positive relationships between the expression of metabolic enzymes and β-catenin were further confirmed in tumour tissues of CRC patients, indicating the pathological significance of the linkage. The *APC* mutation-induced Wnt/β-catenin signalling activation increased the PKM2 and LDHA along with the Warburg effect in CRC cells. The induction of PKM2, a key enzyme mediating the Warburg effect,^20,21^ by *APC* loss occurs via β-catenin/Tcf4-mediated transcription. The role of Wnt/β-catenin signalling in the pathogenesis of CRC related to the Warburg effect is shown by elevation of PKM2 and subsequent increment of the glycolytic genes including *LDHA* and glucose transporter (*GLUT1*) due to *APC* loss. In addition, the critical role of PKM2 in tumour growth induced by *APC*-mutated CRC cells was confirmed in vivo by xenograft mouse model.

These findings provide a key link between aberrantly activated Wnt/β-catenin signalling, especially due to *APC* loss, and the Warburg effect in CRC. In addition, these results suggest that PKM2 could be a potential therapeutic target for CRC caused by aberrant Wnt/β-catenin signalling activation.

## Methods

### Cell culture, transfection and drug treatment

Human CRC cells (DLD1, SW48, SW480, HCT15, HCT116, RKO, and WiDr), U87 cells, and human embryonic kidney (HEK) cell line 293T cells were obtained from the American Type Culture Collection (ATCC, Manassas, VA). DLD1, SW48, SW480, and HCT15 cells were cultured in in RPMI 1640 medium (Gibco) supplemented with 10% foetal bovine serum (FBS; Gibco), and RKO, WiDr, U87, and HEK293T cells were grown in Dulbecco’s modified Eagle’s medium (DMEM; Gibco) containing 10% FBS. HCT116 cells were maintained in McCoy’s 5a Medium (Gibco) containing 10% FBS. Mycoplasma contamination tests were performed for all cells used in this study. KYA1797K was dissolved in dimethyl sulfoxide (DMSO; Sigma-Aldrich) for the in vitro studies. Recombinant wnt3a, EGF and bFGF were purchased from PeproTech.

### 2DE-based proteomics

A total of 3 × 10^6^ DLD1 cells per well were seeded in 100 cm^2^ plates and treated with DMSO or 25 μM KYA1797K for 3 h. The cells were harvested and lysed in the sample buffer (7 M urea, 2 M thiourea, 4.5% CHAPS, 100 mM DTE, 40 mM Tris (pH 8.8)) complemented with complete protease inhibitors (Roche) on ice for 30 min by adding DNase I. The lysates were applied to immobilised pH 3–10 nonlinear gradient strips (Amersham Biosciences) and isoelectric focusing was performed at 80,000 Vh. The second-dimensional separation was performed in 9–16% linear gradient polyacrylamide gels at a constant 40 mA per gel for ∼5 h. After protein fixation in 40% methanol and 5% phosphoric acid for 1 h, the gels were stained with Coomassie brilliant blue G-250 for 12 h. Stained gels were scanned using a GS-710 imaging densitometer (Bio-Rad) and analysed with an Image Master 2-DE Platinum image analysis program (Amersham Biosciences). Expression levels of the spots were determined by the relative spot volume of proteins compared with the volume of a single spot in the gel using the Melanie II program (GenBio).^22^

### Identification of protein spots

For mass spectrometry fingerprinting, protein spots were cut out of the gel and digested using trypsin (Promega), as previously described.^23^ Aliquots of the peptide mixtures obtained from digestion were applied onto a target disk and allowed to air-dry. Spectra were obtained using a Voyager DE PRO MALDI-TOF spectrometer (Applied Biosystems). Protein database searching was performed with MS-Fit^24^ using monoisotopic peaks. A mass tolerance was first allowed within 50 ppm, and then recalibration was performed at 20 ppm after obtaining the protein lists.

### Measurements of glucose and lactate

A total of 5–7 × 10^4^ cells per well were seeded in 12-well plates. Forty-eight hours after plating, the medium was collected, and the glucose and lactate levels were examined using a glucose colorimetric/fluorometric assay kit (Biovision) and lactate colorimetric/fluorometric assay kit (Biovision), respectively, according to manufacturer’s instruction. Glucose and lactate were calorimetrically measured at 590 nm using a FLUOstar OPTIMA (BMG LABTECH), and the glucose consumption and lactate production were normalised to cell number.

### Luciferase reporter assay

The promoter of *PKM2* was subcloned into the pGL3-Basic vector (pGL3; Addgene) to obtain a pGL3-PKM2 promoter-LUC plasmid. The pGL3-PKM2 promoter-LUC plasmid was co-transfected with internal control pCMV-β-galactosidase (β-gal) reporter plasmid (Clontech). When treating Wnt3a proteins, the cells were treated with 50 ng/ml of Wnt3a with fresh media for 24 h after 24 h of transfection. Forty-eight hours after transfection, cells were harvested and lysed in Reporter Lysis Buffer (Promega) according to the manufacturer’s instructions. Luminescence and β-galactosidase activity were measured with a FLUOstar OPTIMA (BMG LABTECH). Luciferase activity was normalised to the β-galactosidase activity. Relative luciferase activity was normalised to the control for each experiment.

### Animal models and analysis of tumour tissue

All animal experiments were performed in accordance with the Korean Food and Drug Administration guidelines. Protocols were reviewed and approved by the Institutional Review Board of Severance Hospital, Yonsei University College of Medicine. C57BL/6J-*Apc*^*Min/+*^ (*Apc*^*min/+*^) mice were obtained from Jackson Laboratory (Bar Harbor, ME). Mice were housed in micro-ventilation cage system (MVCS) cages with a computerised environmental control system (Threeshine Inc.). The temperature was maintained at 24 °C with a relative humidity of 45–55%. Mice were euthanised by carbon dioxide. To generate *Apc*^*min/+*^ mice, *Apc*^*min/+*^ mice were crossed with C57BL/6J^+/+^ (WT) mice. Mouse genotyping was performed using genomic DNA extracted from the tail, and we used male mice for further animal experiments. To control genetic background effects, sex-matched littermates were always used. To investigate the expression level of glycolytic enzymes and β-catenin in WT and *Apc*^*min/+*^ mice, 12-week-old mice were used and were euthanised by carbon dioxide (WT, *n* = 5; *Apc*^*min/+*^, *n* = 6). For the study of in vivo efficacy of KYA1797K, 5-week-old *Apc*^*min/+*^ mice were randomly assigned to 2 groups receiving either vehicle or KYA1797K. The mice were injected i.p. with vehicle (90% PBS and 10% Tween 80; *n* = 6) or KYA1797K (25 mg/kg*; n* = 5) dissolved in the vehicle 5 days per week for 7 weeks. At that time, the mice were euthanised by carbon dioxide and sacrificed. Immediately after sacrifice, the abdomen of each mouse was cut open longitudinally and cleaned by flushing with PBS. Proximal regions of the small intestine were dissected, stained with 0.025% methylene blue (Sigma-Aldrich) for 2 min, and fixed with 4% paraformaldehyde (PFA). Gross images of tumour tissues were captured, and the tissues were resected and embedded in paraffin according to standard procedures. The tumours were classified according to standard World Health Organization histopathological criteria. For histopathologic analyses, a subset of freshly isolated tissues was snap frozen in liquid nitrogen and stored at −80 °C.

### Mouse xenograft assay

All animal experiments were performed in accordance with the Korean Food and Drug Administration guidelines. Protocols were reviewed and approved by the Institutional Review Board of Severance Hospital, Yonsei University College of Medicine. Four-week-old male *athymic BalbC nu/nu* mice were purchased from Joongabio Inc. Mice were housed in micro-ventilation cage system (MVCS) cages with a computerised environmental control system (Threeshine Inc.). The temperature was maintained at 24 °C with a relative humidity of 45–55%. After acclimatisation for 1 week, sixteen mice were randomly divided into two groups and injected subcutaneously in the dorsal flank with 2 × 10^7^ of SW480 stably expressing control shRNA or shRNA targeting PKM2 (shRNA #1) in 100 µl of PBS:Matrigel (BD Bioscience; 1:1) (*n* = 8 per each group) after anesthetised by i.p. injection of sterile avertin (tribromoethanol: 250 mg/Kg). The vital signs of mice were observed each week after injection, and no death was observed in the all groups. Tumours were measured using Vernier callipers, and tumour volume was calculated according to the following formula: π/6 × length × width × height. The mice were euthanised by carbon dioxide when the tumour volume exceeded 1500 mm^3^. Eighty-six days after injection, the mice were sacrificed, and the tumours were excised, weighed, and fixed in 4% PFA or snap frozen in liquid nitrogen for further analysis.

### Tissue microarray

Tissue microarrays (TMA) for normal and cancer tissues, colon disease spectrum tissue array (BC05002a, BC051110b), were purchased from US Biomax. IHC was performed with antibodies against PKM2, LDHA, or β-catenin. From the TMA, 30 normal tissue samples and 104 CRC adenocarcinoma tissue samples were used for further analyses. Signals of the TMA slides were analysed using a bright field microscope (Nikon TE-2000U). For quantitative analysis, the intensity of each staining was determined by IHC Profiler plugin.^25^ All signals were analysed in a double-blind manner.

### Cloning, plasmids and shRNAs

To generate luciferase reporter plasmid containing the promoter of *PKM2*, two DNA fragments (fragment 1, from the upstream region of exon1 to intron1 (−14762 to −10163); fragment 2, from −230 of intron 1 to 5′flanking region of exon2 (21 bp)) were obtained by PCR. The following primers were used: fragment 1, forward 5′-CCTACTATGCACCTAATGTGAGC-3′ and reverse 5′- ACACTTACTGAGTGTGCCACATCC-3′ (including EcoRV restriction site); fragment 2, forward 5′-GTCTAGGTAGATGTCAGTCAGCC-3′ (including SmaI restriction site) and reverse 5′-TTCGAGATGGCTGCTGAGGTCCTGG-3′. The two DNA fragments were ligated and inserted into the pGL3-Basic (pGL3) vector. The cloned plasmid was verified by DNA sequencing (Cosmogenetech). Myc-TCF4-pcDNA3.0 and Myc-ΔN-TCF4E-pcDNA3.0 (dnTCF4) vectors were obtained from Eric R. Fearon of the University of Michigan^26^ and pMD2G and psPAX2 were a gift from Dr. KunLiang Guan, University of California.^27^ β-gal reporter plasmid and pLKO.1puro-β-catenin (β-catenin shRNA) plasmids were purchased from Clontech and Addgene, respectively.

### Lentiviral transduction and stable cell line generation

*APC* knockout (KO) RKO or WiDr cell lines were generated using CRISPR/Cas9 methodology^28^. Briefly, annealed oligonucleotides (CRISPR single guide RNA sequences; sgRNA), targeting the *APC* exon 15 were cloned into the lentiCRISPRv2 (Addgene). The sequences of sgRNA were as follows: sgRNA-1 5′-CACCGTCGCTCTTCATGGATTTTTA-3′; sgRNA-2 5′-AAACTAAAAATCCATGAAGAGCGAC-3′. HEK293T cells were transfected with the lentiCRISPRv2 containing the APC sgRNA and packing vectors pMD2G and psPAX2 at a 2:2:1 ratio for viral production. Then, RKO or WiDr cell lines were transduced with the APC-lentivirus and selected with puromycin (Sigma-Aldrich) to generate the stable cell lines. For generation of *PKM2* knockdown (KD) cell lines, PKM2 shRNAs were designed by Bionics (shPKM2 #1, GCCATCTACCACTTGCAATTA; shPKM2 #2, GCCATAATCGTCCTCACCAAG). *APC* KO-RKO and -WiDr cells were transfected with pGPU6/hygro (shCON) or two independent pGPU6/hygro-PKM2 (shPKM2) vectors, and then selected in a medium containing hygromycin B (1 μg/ml; Duchefa). Of those, PKM2 shRNA #1 was used for real-time PCR analysis, glucose consumption, lactate secretion assays, and mouse xenograft assay. SW480 cells were transfected with the control shRNA or PKM2 shRNA #1 and then selected in a medium containing hygromycin B (1 μg/ml; Duchefa).

### Statistical analysis

All data are expressed as the mean ± standard deviation (s.d.), and the number of samples is indicated in each figure legend. The statistical significance of differences was assessed using the Student’s *t*-test or Spearman correlation analysis. Results shown are representative of at least three independent experiments. Differences reached statistical significance with *P < 0.05, **P < 0.01 and ***P < 0.001. Statistical computations were performed using Prism software (Graph Pad).

## Results

### Expressions of metabolic enzymes are reduced by inhibition of Wnt/β-catenin signalling

**Fig. 1:**
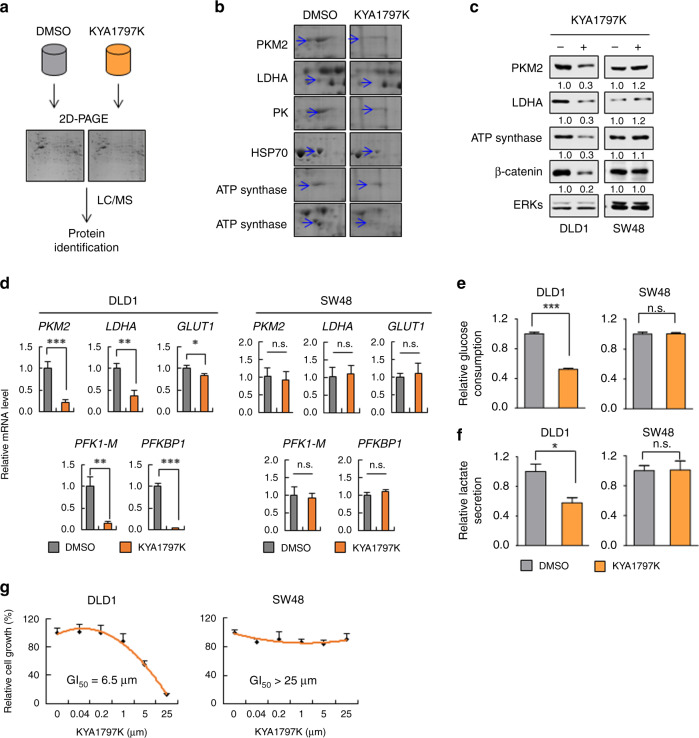
Suppressed expression of metabolic enzymes by KYA1797K. **a** A scheme of 2-DE proteomic analysis for the identification of KYA1797K-regulated proteins in CRC cells. DLD1 cells were treated with DMSO or 25 µM KYA1797K for 3 h, and the cell lysates were subjected to 2-DE proteomic analysis. **b** A zoom-in view of metabolism-related enzymes from 12 spots with distinctly different intensities of 2-DE images. Arrows indicate spots of identified proteins, respectively. **c-f** DLD1 or SW48 cells were treated with DMSO or 25 µM KYA1797K for 24 h. **c** The whole-cell lysates (WCLs) were immunoblotted with the indicated antibodies. **d** The cells were subjected to real-time PCR analyses (*n* = 3). **e**, **f** The relative glucose consumption (**e**) and lactate secretion (**f**) were measured from the collected media from the DLD1 or SW48 cells (*n* = 3). **g** DLD1 or SW48 cells were treated with DMSO or 25 µM KYA1797K for 72 h. Cell proliferation was quantified using the MTT assay (*n* = 3). *P < 0.05, **P < 0.01, ***P < 0.001, n.s. not significant. All data are the mean ± SD. *P* values were determined using the unpaired student’s *t*-test.

In a recent study, we identified KYA1797K, a small molecule that suppresses Wnt/β-catenin signalling by the degradation of β-catenin.^19^ To evaluate the global effects of KYA1797K on cellular proteins related to CRC, we performed proteomic analyses for DLD1 cell extracts by using two-dimensional polyacrylamide gel electrophoresis (2-DE) (Fig. 1a, b and Fig. S1a, b). Twelve proteins, which showed distinct differences in intensities of spots by more than two-fold after treatment with KYA1797K, were identified (Fig. S1a, b). These proteins are known to be involved in various biological processes including glycolysis, cell redox homoeostasis, and the ATP biosynthetic process (Fig. S1b). Among these, 50% (6 out of 12) are proteins involving cancer metabolism (Fig. 1b and Fig. S1a, b); PKM2, LDHA, and pyruvate kinase (PK) are glycolytic enzymes,^29,30^ and stress-70 protein (HSP70) and ATP synthase are associated with the regulation of oxidative phosphorylation.^31^ The reduction of PKM2, LDHA, and ATP synthase by KYA1797K was confirmed in DLD1 cells and several other CRC cell lines (Fig. 1c and Fig. S2). The proteins were not reduced by KYA1797K in SW48 and HCT116 cells expressing non-degradable mutant (MT) β-catenin^32^ (Fig. 1c and Fig. S2), indicating that the regulation of these proteins by KYA1797K is dependent upon β-catenin destabilisation. The β-catenin-dependent decrease in the glycolytic enzymes by KYA1797K occurs at the level of transcription, as shown by the measurement of their mRNA levels (Fig. 1d). Consistent with their gene expression levels, both glucose consumption and lactate secretion were reduced by KYA1797K treatment, and these phenomena were observed only in DLD1 cells but not in SW48 cells (Fig. 1e, f). In addition, the inhibition of cell proliferation by KYA1797K occurred in DLD1 but not in SW48 cells (Fig. 1g).

### Expressions of glycolytic enzymes are positively correlated with β-catenin in human CRCs

**Fig. 2:**
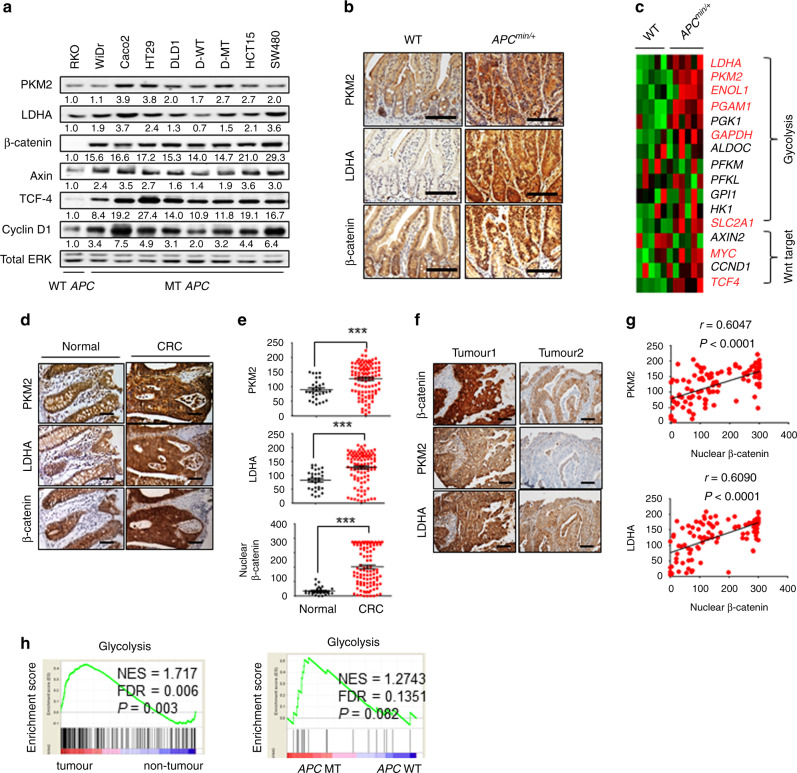
Positive correlation of PKM2 and LDHA expression with Wnt/β-catenin signalling in CRCs. **a** The WCLs of normal human colonic fibroblast CCD18-Co and 9 CRC cells (RKO, WiDr, CaCo2, HT29, DLD1, D-WT, D-MT, HCT15, and SW480) were immunoblotted with the indicated antibodies. **b** IHC analysis of small intestine tissue from WT mice and adenomas from *Apc*^*min/+*^ mice (WT, *n* = 5; *Apc*^*min/+*^, *n* = 6). Scale bars represent 50 μm. **c** Heat map of gene expression for glycolysis genes and Wnt signalling target genes of normal and adenoma small intestine tissues from WT and *Apc*^*min/+*^ mice, respectively (dataset; GSE422). Genes significantly upregulated in *Apc*^*min/+*^ mice are indicated in red (*P* < 0.05 using Student’s *t*-test). **d–g** IHC images and the corresponding quantification data for CRC TMA samples (normal, *n* = 30; CRC, *n* = 104) were performed. **d** Representative IHC analyses of PKM2, LDHA, and β-catenin abundance in normal and cancer tissues of TMA samples. Scale bars represent 50 μm. **e** IHC Profiler was used for quantitative analysis for the staining of PKM2, LDHA, and nuclear β-catenin. **f** Representative images of four CRC samples from the IHC staining for 104 CRC tissues of the TMA samples. Scale bars represent 50 μm. **g** Spearman correlation analysis for nuclear β-catenin with PKM2 and LDHA levels in 104 CRC tissues. **h** GSEA of human CRCs (data set; GSE8671) comparing glycolysis gene sets in adenoma from 32 CRC patients and corresponding normal tissues from the same individuals (left panel). GSEA of human CRCs (data set; GSE26906) comparing glycolysis gene sets in CRC patients with and without *APC* mutations (right panel). NES, normalised enrichment score; FDR false discovery rate. ***P < 0.001. *P* values were determined using the unpaired student’s *t*-test.

To investigate the pathophysiological relevance of the relationship between expression of glycolytic enzymes and Wnt/β-catenin signalling, we first evaluated the expression of β-catenin, PKM2, and LDHA in various CRC cell lines (Fig. 2a). As expected, the β-catenin is overexpressed in most CRC cells containing MT *APC* compared with those in CRC cells harbouring WT *APC* (Fig. 2a). The protein levels of the Wnt/β-catenin signalling target genes, Axin, Cyclin D1, and TCF-4, were also increased with some individual variations in the CRC cells harbouring *APC* mutations (Fig. 2a). Expression of PKM2 and LDHA showed a high positive correlation with the β-catenin level in CRC cells (Fig. 2a). To examine the correlation of the expression of PKM2 and LDHA with Wnt/β-catenin signalling, we investigated their expression in small intestine tissue samples from WT and *Apc*^*min/+*^ mice.^33^ PKM2 and LDHA levels were higher in tumours from *Apc*^*min/+*^ mice compared with those from WT mice (Fig. 2b). Gene ontology analyses of data from WT and *Apc*^*min/+*^ mice showed that the genes associated with the metabolic process were significantly increased in small intestine tumour tissues from *Apc*^*min/+*^ mice compared with those in small intestine tissues of WT mouse (Fig. S3a). Especially, the expressions of glycolytic genes were significantly up-regulated together with Wnt target genes in *Apc*^*min/+*^ mice compared with those in WT mice (Fig. 2c).

To further investigate the clinical relevance of PKM2 and LDHA expression related to β-catenin in CRC, we then analysed their expression in a human colorectal tissue microarray (TMA). PKM2 and LDHA were elevated with β-catenin in 70.2% (73/104) of CRCs compared with normal colon tissues (Fig. 2d, e). Furthermore, the levels of PKM2 and LDHA were positively correlated with that of nuclear β-catenin (Fig. 2f). The H-score quantification of the staining revealed the significance of the correlation among expression of PKM2 and LDHA with nuclear β-catenin (Fig. 2g). The validated human CRC patient microarray data sets showed that the expressions of glycolytic genes including *PKM* and *LDHA* were co-increased with Wnt/β-catenin signalling target genes such as c*-Myc*, *CCND1* (encoding Cyclin D1), and *AXIN2* in CRCs compared to those expressed in adjacent normal tissues (Fig. S3b). Gene set enrichment analysis (GSEA) also revealed that glycolytic genes are enriched in CRC (Fig. 2h; left panel). Furthermore, when classifying CRC patients with and without *APC* mutations, the glycolytic genes are enriched in CRC with *APC* mutations (Fig. 2h; right panel). Altogether, expression of the glycolytic enzymes PKM2 and LDHA are highly increased and showed a positive correlation with β-catenin in CRC.

### PKM2 and LDHA expression and the Warburg effect are controlled by Wnt/β-catenin signalling

**Fig. 3:**
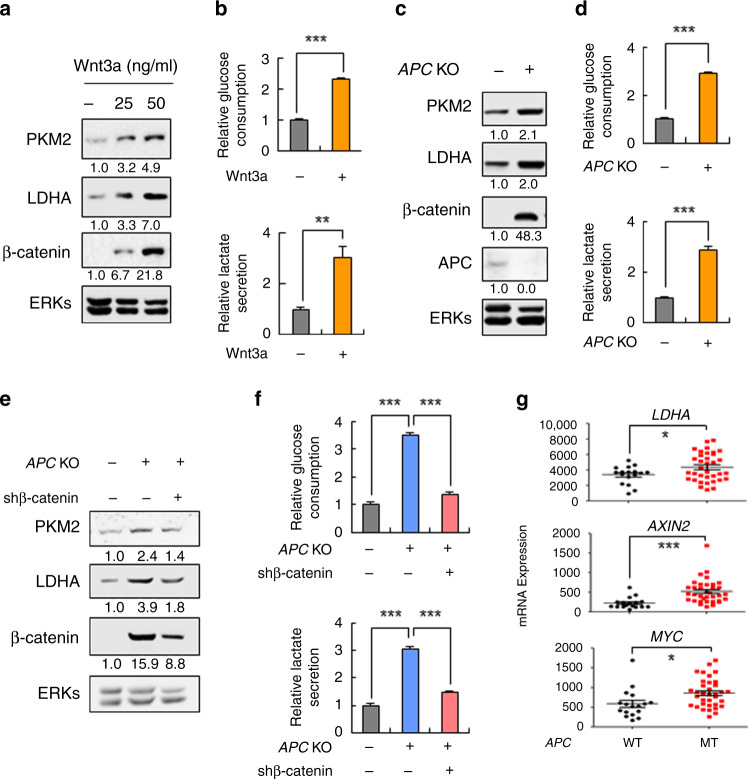
Regulation of PKM2 and LDHA expression and the Warburg effects by the Wnt/β-catenin signalling in CRC cells. **a**, **b** RKO cells were treated with recombinant Wnt3a proteins for 24 h. Immunoblotting (**a**) and the relative glucose consumption and lactate secretion were measured (**b**) (*n* = 3). The relative glucose consumption and lactate secretion were measured from the collected media, and the values were normalised to the values from the control. **c**, **d** RKO cells harbouring the WT or KO *APC* gene were harvested 24 h after seeding. Immunoblotting of the WCLs (**c**) and the relative glucose consumption and lactate secretion assay were performed (**d**) (*n* = 3). **e**, **f** *APC* WT-RKO, *APC* KO-RKO cells were transfected with pLKO.1puro (control) or pLKO.1puro-β-catenin (β-catenin shRNA) plasmids. After 48 h, immunoblotting (**e**) and measurement of the relative glucose consumption and lactate secretion (**f**) were performed (*n* = 3). The relative glucose consumption and lactate secretion were measured from the collected media, and the values were normalised to the values from *APC* WT-RKO cells. **g** Gene expression of *LDHA, AXIN2*, and *MYC* in human CRC samples harbouring WT and MT *APC* (WT, *n* = 17; cancer, *n* = 35; data set, GSE63624). *P < 0.05, **P < 0.01, ***P < 0.001. All data are the mean ± SD. *P* values were determined using unpaired student’s *t*-test.

To explore the role of Wnt/β-catenin signalling in the expression of glycolytic enzymes, the effects of recombinant Wnt3a were investigated by using RKO cells. Both protein and mRNA levels of PKM2 and LDHA were increased by treatment with Wnt3a (Fig. 3a and Fig. S4a). The mRNA levels of *GLUT1*, *PFK1-M*, and *PFKBP1* were similarly increased by Wnt3a treatment (Fig. S4a). The glucose consumption and lactate secretion were also significantly augmented by Wnt3a treatment in RKO cells (Fig. 3b). The expression of glycolytic enzymes including PKM2 and LDHA as well as the glucose consumption and lactate secretion were similarly increased with the activation of β-catenin by *APC* knockout in RKO or WiDr cells (Fig. 3c, d and Fig. S4c-e). The co-increment of PKM2/LDHA and β-catenin with that of glucose consumption and lactate secretion by *APC* knockout were all lowered by β-catenin knockdown (Fig. 3e, f and Fig. S4f, g), confirming the role of Wnt/β-catenin signalling in the regulation of PKM2 and LDHA expression and the Warburg effect. Furthermore, the changes in the expression of *LDHA, MYC*, and *AXIN2* due to *APC* mutation were further supported by analyses of human CRC microarray data sets (Fig. 3g). Overall, the expression of the glycolytic enzymes and subsequently the Warburg effect were controlled by Wnt/β-catenin signalling in CRC cells.

### The induction of glycolytic enzymes in the small intestine of *Apc*^*min/+*^ mice was suppressed by the Wnt/β-catenin signalling inhibitor KYA1797K

**Fig. 4:**
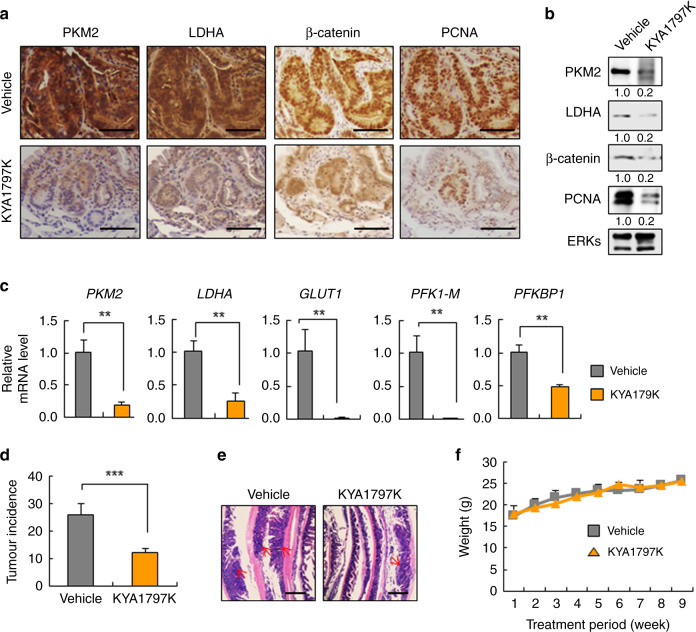
Suppression of induced glycolytic enzymes by KYA1797K in *Apc*^*min/+*^ mice. **a–f** Five-week-old *Apc*^*min/+*^ mice were intraperitoneally injected with vehicle or KYA1797K (25 mg/kg) 5 days per week for 7 weeks. **a** IHC analysis of tumour tissues treated with vehicle or KYA1797K. Scale bars represent 50 μm. **b** WCLs prepared from tumour tissues were subjected to immunoblotting analyses. **c** Real-time PCR analyses were performed for the tumour tissues (*n* = 3). **d** The total incidence of tumours was plotted (Vehicle, *n* = 6; KYA1797K, *n* = 5). **e** Swiss roll images from the proximal areas containing the duodenum of the small intestine (H&E staining). Red arrows indicate tumours and scale bars represent 400 μm. **f** The weight of each mouse was measured every 4 days. (Vehicle, *n* = 6; KYA1797K, *n* = 5). **P < 0.01, ***P < 0.001. All data are the mean ± SD. *P* values were determined using unpaired student’s *t*-test.

To investigate whether β-catenin destabilisation could decrease the expression of glycolytic enzymes induced by *APC* mutation in vivo, KYA1797K was applied to *Apc*^*min/+*^ mice, which display increased tumour incidences in small intestines with Wnt/β-catenin signalling activation. The expression levels of PKM2 and LDHA were significantly decreased together with β-catenin and proliferating cell nuclear antigen (PCNA) by KYA1797K treatment in the tumour area of the small intestine from *Apc*^*min/+*^ mice compared with those of vehicle-treated mice (Fig. 4a, b). The regulation of glycolytic enzyme expression by Wnt/β-catenin signalling was also supported by reductions of mRNA levels of the enzymes in KYA1797K-treated tumour tissues (Fig. 4c). Furthermore, inhibition of Wnt/β-catenin signalling by KYA1797K reduced tumour incidence in the small intestine of *Apc*^*min/+*^ mice (Fig. 4d, e) without a significant change in body weight (Fig. 4f). Overall, these results showed that increased glycolytic enzyme expression and tumour growth due to *APC* loss were diminished by inhibition of Wnt/β-catenin signalling in vivo. Overall, expressions of the glycolytic enzymes were increased together with tumours by *Apc* loss in vivo, and these were diminished by the small molecular inhibition of Wnt/β-catenin signalling.

### Wnt/β-catenin signalling regulates transcription of *PKM2* via Tcf4

**Fig. 5:**
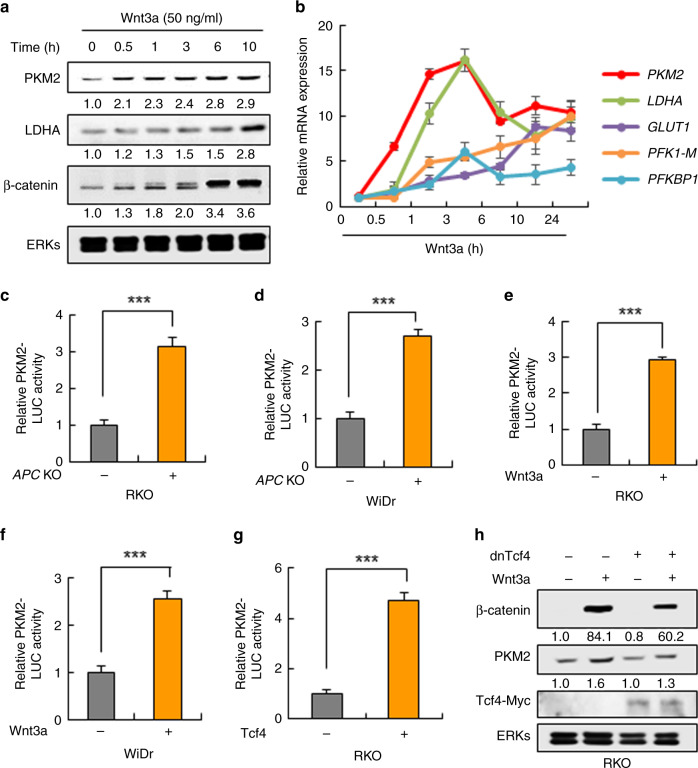
Regulation of *PKM2* transcription by Wnt/β-catenin signalling via Tcf4 in CRC cells. **a**, **b** RKO cells were treated with 50 ng/ml of Wnt3a for various time periods, and the cells were subjected to immunoblotting with the indicated antibodies (**a**) and real-time PCR analyses (**b**) (*n* = 3). **c–g** The cells were transfected with PKM2 promoter-contained pGL3 luciferase reporter plasmid (PKM2 reporter) and pCMV–β-galactosidase (β-gal) as an internal control. **c**, **d** Luciferase activities were assayed 48 h post-transfection of PKM2 reporter and β-gal (*n* = 3). **e**, **f** RKO (**e**) or WiDr (**f**) cells were treated with 50 ng/ml of Wnt3a, EGF, or bFGF for 24 h after 24 h of the transfection. Then, the cells were subjected to the luciferase reporter assay (*n* = 3). **g** RKO cells were transfected with pcDNA3.0 or Myc-TCF4-pcDNA3.0 and PKM2 reporter and β-gal. After 48 h, the cells were subjected to the luciferase assay (*n* = 3). **h** RKO cells were transfected with pcDNA3.0 or Myc-ΔN-TCF4E-pcDNA3.0 (dnTCF4) vector. After 24 h, the cells were treated with 50 ng/ml Wnt3a for 24 h and immunoblotted with indicated antibodies. ***P < 0.001. All data are the mean ± SD. *P* values were determined using the unpaired student’s *t*-test.

We evaluated expression profiles of the glycolytic enzymes using Wnt3a in RKO cells during various time points to investigate how activated Wnt/β-catenin signalling increases glycolytic enzyme expression. PKM2 was increased together with β-catenin at 30 min after Wnt3a treatment, but the change in other glycolytic genes including *LDHA* was shown at later time points than that of *PKM2* (Fig. 5a, b). Next, because the *PKM2* gene has putative binding sites for the T-cell factor (Tcf4/TCF7L2^34^) on its promoter, we tested its involvement in the transcriptional regulation of *PKM2*. The transcriptional activity of the *PKM2* reporter harbouring the genomic DNA upstream of the transcription start site of the *PKM2* was increased by *APC* KO (Fig. 5c, d) in both RKO and WiDr cells. The reporter activity was similarly increased by Wnt3a treatment in both RKO and WiDR cells (Fig. 5e, f), and that was also augmented by Tcf4 overexpression (Fig. 5g). The β-catenin/Tcf4-dependent transcriptional induction of *PKM2* was confirmed by the abolishment of β-catenin induction by overexpression of the dominant negative (DN)-Tcf4 (Fig. 5h). Since expression and nuclear localisation of PKM2 is regulated by epidermal growth factor (EGF) in U87 human glioblastoma cells,^35^ we tested the effects of EGF and also basic fibroblast growth factor (bFGF), a clinically important growth factor in CRCs, on PKM2 expression in CRC cells. The induction of PKM2 was specific to Wnt/β-catenin signalling in RKO CRC cells (Fig. S5a, b). Overall, *PKM2* was specifically induced by Wnt/β-catenin signalling through transcriptional activation by Tcf4 in CRC cells.

### PKM2 mediates the Wnt/β-catenin signalling-induced Warburg effect and tumorigenesis in CRC

**Fig. 6:**
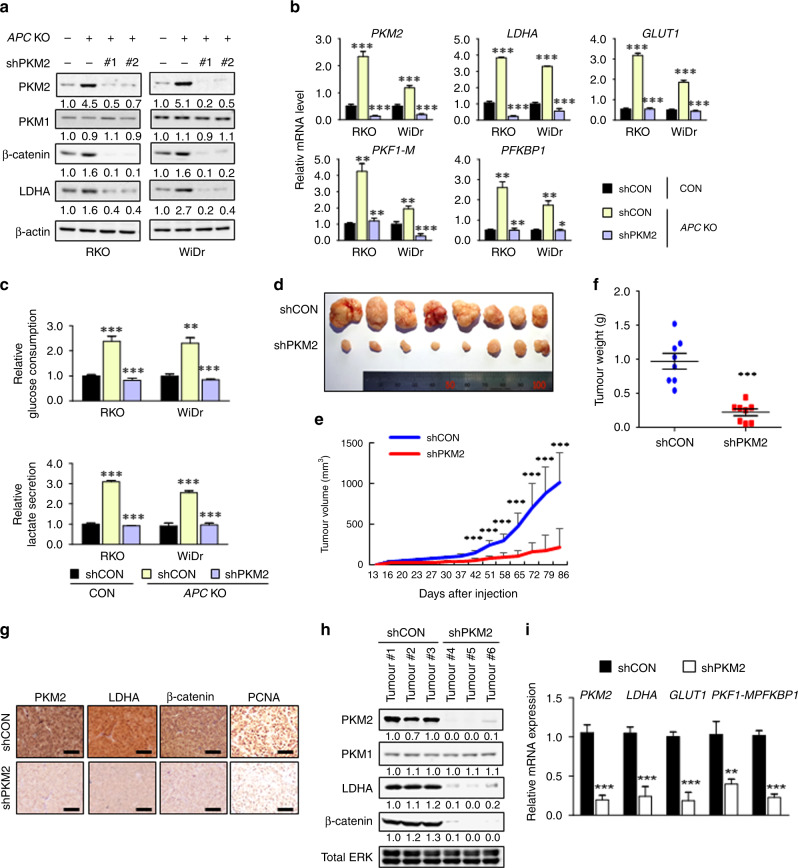
Roles of PKM2 on the Wnt/β-catenin signalling-induced Warburg effect and tumorigenesis in CRC. **a** Immunoblot analyses of control-RKO, *APC* KO-RKO, *APC* KO-RKO shPKM2 #1, and *APC* KO-RKO shPKM2 #2 cells (left panel) and control-WiDr, *APC* KO-WiDr, *APC* KO-WiDr shPKM2 #1, and *APC* KO-WiDr shPKM2 #2 cells (right panel). WCLs were prepared and probed with antibodies recognising the indicated proteins. **b** Glycolytic gene expression levels were analysed using real-time PCR analyses in control-RKO, *APC* KO-RKO, and *APC* KO-RKO shPKM2 #1 cells (left part of the graph) and control-WiDr, *APC* KO-WiDr, and *APC* KO-WiDr shPKM2 #1 cells (right part of the graph) (*n* = 3). **c** The media collected from control-RKO, *APC* KO-RKO, and *APC* KO-RKO shPKM2 #1 cells (left part of the graph) and control-WiDr, *APC* KO-WiDr, and *APC* KO-WiDr shPKM2 #1 cells (right part of the graph) were assayed for measurement of relative glucose consumption and lactate secretion. The values were normalised to control-RKO (left part of the graph) or control-WiDr (right part of the graph) (*n* = 3). **d–i** SW480 cells harbouring *APC* mutation were injected subcutaneously into nude mice (*n* = 8). **d** Representative images show respective xenograft tumours at day 86 post subcutaneous injection. **e**, **f**)Tumour volumes were measured throughout the experiment (**e**) and tumour weight was measured at the time of sacrifice (**f**). Data represent the mean ± s.d. (eight mice per group). **g** IHC analyses for the indicated proteins from tumours of control cells and PKM2 knockdown cells. Scale bars represent 100 μm. Immunoblot analyses for the indicated proteins (**h**) and qRT-PCR analyses of glycolytic genes (**i**) in tumours of control cells and PKM2 knockdown cells. **P < 0.01, ***P < 0.001. All data are the mean ± SD. *P* values were determined using the unpaired student’s *t*-test.

Given that the β-catenin-Tcf4 axis increased the PKM2 level in CRC cells, we investigated the roles PKM2 on the Wnt/β-catenin signalling-induced Warburg effect in CRCs. PKM2, but not PKM1, knockdown significantly reduced the PKM2 and LDHA inductions, which were shown in the *APC* KO-RKO and -WiDr cells (Fig. 6a). In addition, the enhancement of glycolytic enzyme expression, glucose consumption, and lactate secretion in the *APC* KO-RKO and -WiDr cells was also suppressed by PKM2 knockdown (Fig. 6b, c). Similarly, the induction of PKM2 and LDHA as well as the increments of the glycolytic enzyme expression, glucose consumption, and lactate secretion by Wnt3a treatment were inhibited by PKM2 knockdown (Fig. S6a–c). Overall, PKM2 is a critical mediator in the induction of glycolytic enzymes and the Warburg effect by Wnt/β-catenin signalling activation.

To determine the role of PKM2 in tumour development promoted by *APC* mutation-induced Wnt/β-catenin signalling activation, we subcutaneously injected *APC*-mutated SW480 CRC cells stably expressing the control shRNA or PKM2 shRNA into nude mice. Both the volume and weight of tumours were significantly decreased in *APC*-mutated CRC cells stably expressing PKM2 shRNA compared with those of *APC*-mutated CRC cells containing control shRNA (Fig. 6d–f). LDHA, β-catenin, and PCNA increased in the tumour tissues induced by injection of *APC*-mutated CRC cells expressing control shRNA and were significantly reduced in those generated by injection of the identical cells expressing PKM2 shRNA, as shown by IHC (Fig. 6g) and western blot analyses (Fig. 6h). Similarly, the mRNA levels of the glycolytic enzymes were mostly abolished in PKM2 knockdown SW480 cells (Fig. 6i). Overall, PKM2 is a critical mediator for the Wnt/β-catenin signalling-induced Warburg effect and tumour growth in CRCs.

## Discussion

The Wnt/β-catenin signalling pathway plays a role in the metabolic homoeostasis of normal cells as well as carcinogenesis of several different cancer types, and its dysregulation is closely related to metabolic disorders such as obesity and diabetes.^36–38^ However, different from normal cell metabolism, the role of the Wnt/β-catenin signalling pathway in altered cancer metabolism is poorly understood.

In this study, we identified that PKM2 and LDHA, the key players in the Warburg effect, were significantly suppressed by the Wnt/β-catenin signalling inhibitor KYA1797K^19^ in CRC cells, and that led us to identify a role of Wnt/β-catenin signalling, especially its aberrant activation due to *APC* loss, in cancer metabolism and the Warburg effect. *PKM2* and *LDHA* were transcriptionally induced along with the Warburg effect by activation of Wnt/β-catenin signalling, and this was shown by monitoring the effects of a Wnt ligand or genetic modulations of the Wnt/β-catenin signalling as well as by showing the role of β-catenin/Tcf4 on the promoter of *PKM2*. The pathological significance of the aberrantly activated Wnt/β-catenin signalling on the Warburg effect was indicated by a positive correlation between PKM2/LDHA and β-catenin expressions in the tumour tissues of *Apc*^*min/+*^ mice and CRC patients harbouring *APC* mutations as high as 90%.^13^

PKM2 is frequently overexpressed in various cancers including CRCs, and its expression correlates with tumorigenesis.^29,39^ We identified that PKM2 plays a key role in the Wnt/β-catenin signalling-dependent activation of glycolytic genes and the Warburg effect in CRC. Therefore, the induction of other glycolytic genes such as *LDHA, GLUT1*, *PFK1-M*, and *PFKBP1* as well as the Warburg effect by *APC* loss were all critically suppressed by PKM2 knockdown. Moreover, the significance of PKM2 in the tumorigenesis induced by aberrantly activated Wnt/β-catenin signalling was confirmed by using xenograft model system with *APC* mutated*-*CRC cells stably expressing control shRNA or PKM2 shRNA.

PKM2 also reported to be regulated by EGF or EGFR, as shown by its upregulation and translocation into the nucleus followed by interaction with β-catenin for cell proliferation in brain tumours.^35,40^ However, in CRC cells, PKM2 is specifically upregulated by Wnt3a, and not EGF or bFGF. These results indicate that the Wnt/β-catenin signalling-mediated regulation of cancer metabolism and the Warburg effect may be specific to CRC cells. In addition, PKM2 is known to be transcriptionally regulated by c-Myc, a well-known Wnt/β-catenin signalling target gene regulating the cancer metabolism.^41^ In addition, positive feedback regulations between PKM2 and c-Myc in the Warburg effect have been elucidated.^29^ However, we did not observe any noticeable correlation of its expression with PKM2 in various CRC cells. In addition, the expression of c-Myc was not correlated with Wnt/β-catenin signalling in several CRC cell lines (data not shown). The results may be attributed to the fact that the expression of c-Myc was affected by various mitogenic signals such as Sonic hedgehog (Shh) and EGF^42^ as well as Wnt/β-catenin signalling and other unknown factors. Therefore, we explored TCF-4, shown the correlation with PKM2 and β-catenin, as a mediator between PKM2 and the Wnt/β-catenin signalling in the Warburg effect of CRCs.

Most of our study focused on the regulation of PKM2 via Wnt/β-catenin signalling; however, we found that the Wnt/β-catenin pathway was also subjected to regulation via PKM2, as indicated by the decrease of β-catenin by PKM2 knockdown. Therefore, a positive loop that accelerates the activation of Wnt/β-catenin signalling and PKM2 expression could be present in the tumorigenesis accompanying cancer metabolism and the Warburg effect in colorectal tumorigenesis. On the basis of the results described in this study, we suggest the following model (Fig. S7) for the regulation of *APC* mutation-induced Wnt/β-catenin signalling in the Warburg effect and tumorigenesis in CRCs: *APC* mutation increases β-catenin and enhances the β-catenin/Tcf4 binding on *PKM2* promoter regions, which subsequently augments its transcription. The increased PKM2 further enhances the expression of other glycolytic enzymes and induces the Warburg effect together with the reactivation of β-catenin signalling in colorectal tumorigenesis.

Overall, our study suggests that aberrant activation of Wnt/β-catenin signalling such as by pathologically important *APC* mutation directs cancer metabolism and the Warburg effect via PKM2 in CRC. Therefore, targeting cancer metabolism, especially the key factor PKM2, could be an ideal approach for the treatment of CRC.

## Supplementary information

Supplementary information

## Data Availability

The datasets generated and/or analysed during the current study are available through Gene expression omnibus (GEO) or the corresponding references. Data of the microarray analysis: Gene Expression. Data of the microarray analysis on normal tissues and adenoma of small intestine tissues from WT and *Apc*^*min/+*^ mice are shown in Fig. 2c and Fig. S3a: GSE422. Data of the microarray analysis on human normal colon and CRC samples are shown in Fig. S3b: GSE9348. Enrichment of glycolysis and glyconeogenesis in human tissues of normal mucosa and colorectal adenomas is shown in Fig. 2h (left panel): GSE8671. Enrichment of glycolysis and glyconeogenesis in CRC patients with and without *APC* mutations is shown in Fig. 2h (right panel): GSE26906. Gene expression of *LDHA, AXIN2* and *MYC* in human CRC samples harbouring WT and MT *APC* in Fig. 3g: GSE63624.
