# Supplementary material for: APC loss induces Warburg effect via increased PKM2 transcription in colorectal cancer
Source: Br J Cancer. 2020 Oct 19;124(3):634–44. doi: 10.1038/s41416-020-01118-7 (PMC7851388; doi:10.1038/s41416-020-01118-7)
Supplement: Supplementary file 1 — Supplementary information [file 41416_2020_1118_MOESM1_ESM.docx]

***APC* loss induces Warburg effect via increased *PKM2* transcription in colorectal cancer**

**Pu-Hyeon Cha^1^**^†^**, Jeong-Ha Hwang^1^**^†^**, Dong-Kyu Kwak^1^, Eunjin Koh^2^, Kyung-Sup Kim^2^ and Kang-Yell Choi^1,3*^**

^1^Department of Biotechnology, College of Life Science and Biotechnology, Yonsei University, Seoul, Korea

^2^Department of Biochemistry and Molecular Biology, Integrated Genomic Research Center for Metabolic Regulation, Institute of Genetic Science, College of Medicine, Yonsei University, Seoul, Korea.

^3^CK Biotechnology Inc., Building 117, 50 Yonsei Ro, Seodaemun-Gu, Seoul, Korea.

^†^ These authors contributed equally to this work.

*Correspondence: Dr. KY Choi, E-mail: [kychoi@yonsei.ac.kr](mailto:kychoi@yonsei.ac.kr)

**Supplementary Methods**

**Immunoblotting**

After washing with ice-cold PBS (Gibco), cells were lysed with radio-immunoprecipitation assay (RIPA) buffer (Millipore). Tumor samples of mice tissues were stored in liquid nitrogen and were homogenized in RIPA buffer for preparation. Protein samples were loaded in a 6–15% sodium dodecyl sulfate (SDS) polyacrylamide gel and transferred to a nitrocellulose membrane (Whatman). The following primary antibodies were used for immunoblotting: anti-β-catenin (Santa Cruz Biotechnology; sc-7199; 1:500-1:3000), anti-PKM2 (Cell Signaling Technology; #3198; 1:15000), anti-LDHA (Santa Cruz Biotechnology; sc-137243; 1:15000), anti-ATP synthase (Novus Biologicals; NBP1-31242; 1:1000), anti-ERK (Santa Cruz Biotechnology, sc-514302; 1:5000), anti-PCNA (Santa Cruz Biotechnology, sc-56; 1:1000), anti-c-Myc (Santa Cruz Biotechnology, sc-789; 1:1000), anti-Tcf4 (Santa Cruz Biotechnology, sc-166699; 1:1000), anti-PKM1 (Cell Signaling Technology; #7067; 1:3000), anti-APC (Santa Cruz Biotechnology, sc896; 1:1000), and anti-GFP (Applied Biological Materials INC., G096; 1:3000). Secondary antibodies, horseradish peroxidase-conjugated anti-mouse (Cell Signaling Technology, #7076; 1:3000) or anti-rabbit (Bio-Rad, #1706515; 1:3000) were used in this study.

**Reverse transcription and quantitative real-time PCR**

Total RNA was extracted using TRIzol reagent (Invitrogen) according to the manufacturer’s instructions. The cDNA was prepared with total RNA (2 µg) and 200 units of reverse transcriptase (Invitrogen) in a 20-µl reaction carried out at 37°C for 1 h. The resulting cDNA (1 μl) was amplified in 10 μl of iQ SYBR Green Supermix (Bio-Rad, Hercules, CA). The comparative cycle-threshold (CT) method was used, and the data were normalized to the expression of β-actin. The experiment was performed by three independent experiments in triplicate. The primer sets were as follows: GLUT1, forward 5′-CGGGCCAAGAGTGTGCTAAA-3′ and reverse 5′-TGACGATACCGGAGCCAATG-3′; LDHA, forward 5′-ATCTTGACCTACGTGGCTTGGA-3′ and reverse 5′-CCATACAGGCACACTGGAATCTC-3′; PKM2, forward 5′-TGGCGCCCATTACCAGCGACC-3′ and reverse 5′-TCCCTTCTTGAAGAAGCCTCGGGCCTT-3′; PFK1-M, forward 5′- GAGTGACTTGTTGAGTGACCTCCAGAAA-3′ and reverse 5′-TCCCTTCTTGAAGAAGCCTCGGGCCTT-3′; PFKBP1, forward 5′- GCATCCCATTTGTGGTCATTC-3′ and reverse 5′-GTCACAGGTTGTGCAGATAGT-3′; CTNNB1 (which encodes β-catenin), forward 5′-ACAAGCCACAAGATTACAAGAA-3′ and reverse 5′-GCACCAATATCAAGTCCAAGA-3′; ACTB (for β–actin), forward 5′-ATAGCACAGCCTGGATAGCAAC-3′ and reverse 5′-AATCTGGCACCACACCTTCTAC-3′.

**Cell proliferation assay**

DLD1 or SW48 cells were plated at a density of 5 × 10^3^ cells/well in a 96-well plate. The cells were then treated with 0.04, 0.2, 1, 5, or 25 µM KYA1797K or control (DMSO) for 96 h. Next, 3-(4,5-dimethylthiazol-2-yl)-2-5-diphenyltetrazolium bromide (MTT; AMRESCO) reagent was added to each well at a concentration of 0.25 mg/ml. After incubation for 2 h at 37°C, insoluble purple formazan was obtained by removing the medium and incubating in 200 μl (96-well) of DMSO for 1 h. The absorbance of the formazan product was determined at 590 nm using a FLUOstar OPTIMA (BMG LABTECH). The relative cell growth was normalized to the control (DMSO).

**Immunohistochemistry (IHC)**

Hematoxylin and eosin (H&E) staining for tissue sections was performed according to standard procedures ^25^. For IHC analysis, 4 µm paraffin-embedded tissue sections were treated with citrate buffer (pH 6.0) and autoclaved for 15 min. To block endogenous peroxidase activity prior to peroxidase IHC analysis, tissues were incubated with 0.345% H_2_O_2_ (Samchun Chemicals) for 30 min. The sections were then blocked with 5% bovine serum albumin (BSA) and 1% normal goat serum (NGS; Vector Laboratories) in PBS for 30 min or in 10% BSA and 1% NGS in PBS for 1 h for mouse tumor samples. For peroxidase IHC analysis, before incubating sections with mouse primary antibody, mouse IgG was blocked using a M.O.M Mouse IgG blocking kit (Vector Laboratories). Sections were incubated with primary antibody overnight at 4°C, followed by incubation with biotinylated anti-mouse (Dako, E-0433; 1:300) or biotinylated anti-rabbit (Dako, E-0353; 1:300) secondary antibodies for 1 h at room temperature. The following primary antibodies were used for the IHC: anti-β-catenin (BD Bioscience, #610154; 1:200), anti-PKM2 (Cell Signaling Technology; #3198; 1:2000 for mouse, 1:500 for human), anti-LDHA (abcam; ab84716; 1:200), and anti-PCNA (Santa Cruz Biotechnology, sc-56; 1:200). The samples were then incubated in avidin-biotin complex solutions (Vector Laboratories), stained with 3, 3'-diaminobenzidine (DAB; Dako) for 3−7 min and counterstained with Mayer’s hematoxylin (Muto). All incubations were conducted in humid chambers. Signals were analyzed using a bright field microscope (Nikon TE-2000U).

**
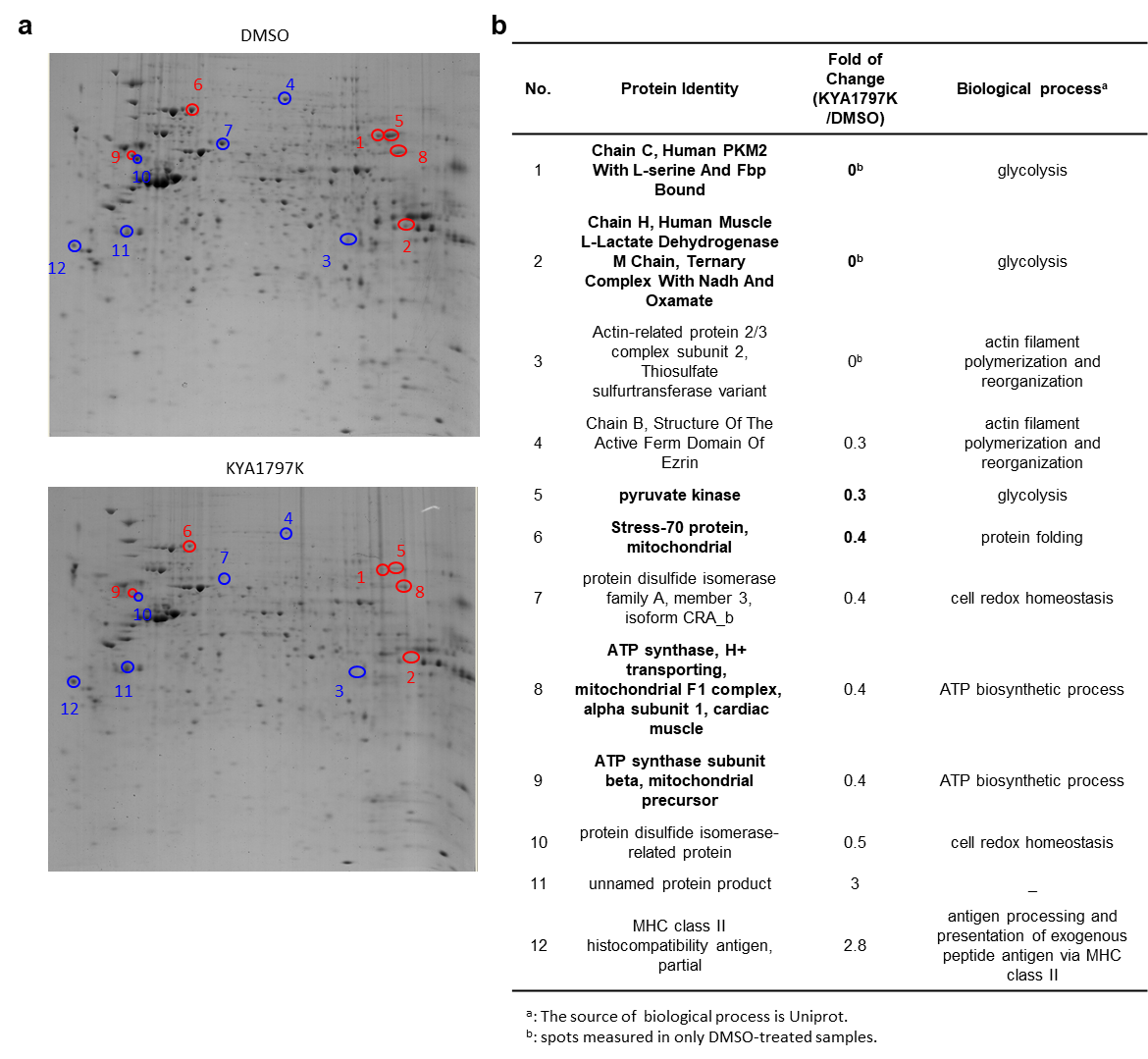
Supplementary Figures 1-6**

**Fig. S1. Investigation of global effects of KYA1797K on proteins using 2-DE proteomic analysis.** **a**-**b** 2-DE proteomic analysis was performed for DLD1 cells treated with DMSO or 25 µM KYA1797K for 3 h. Twelve proteins with intensity changes of more than two-fold as a ratio of DMSO levels were identified by LC/MS analysis. (**a**) Whole gel images for the 2-DE proteomic analysis. In the 12 identified proteins, metabolism-related proteins and non-metabolism-related protein were marked with red and blue circles, respectively. (**b**) List of identified 12 proteins including fold changes and their biological process. Metabolism-related proteins are indicated by bold.

**
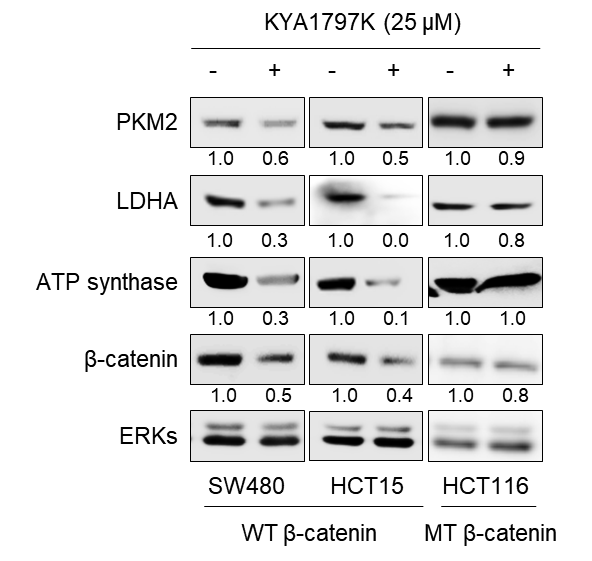
**

**Figure S2. β-Catenin-dependent regulation of metabolism enzyme expression by KYA1797K in CRC cells.** SW480, HCT15, or HCT116 cells were treated with DMSO or 25 µM KYA1797K for 24 h, and the whole cell lysates (WCLs) were immunoblotted with the indicated antibodies.


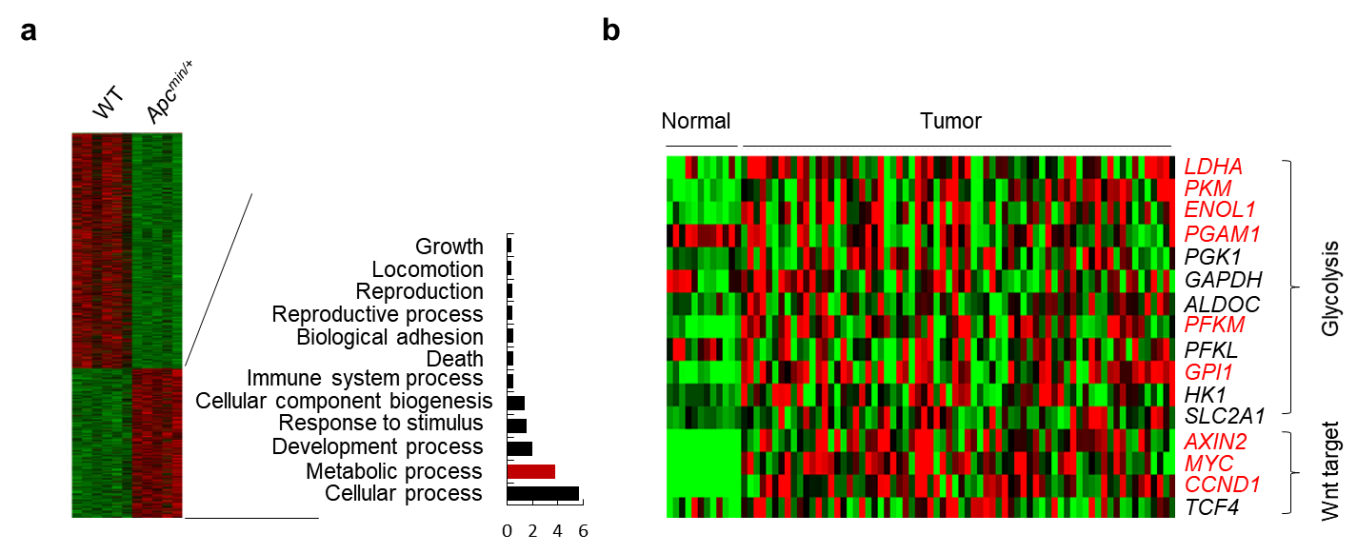


**Fig. S3. Gene ontology analyses and comparison of expression for glycolysis and Wnt target gene in mouse and human CRC model samples. a** Heat map illustrating differential gene expression between normal and adenoma of small intestine tissues from WT and *Apc^min/+^* mice (dataset; GSE422), respectively (left). Functional categorization of upregulated genes in *Apc^min/+^* mice samples compared with WT mouse samples (right) **b** Heat map of gene expression for glycolysis genes and Wnt signaling target genes of normal (*n* = 12) and tumor (*n* = 70) samples from human CRC patients (dataset; GSE9348). Significantly upregulated genes in the tumor samples are marked in red (*P* < 0.05 using Student’s *t*-test).

**
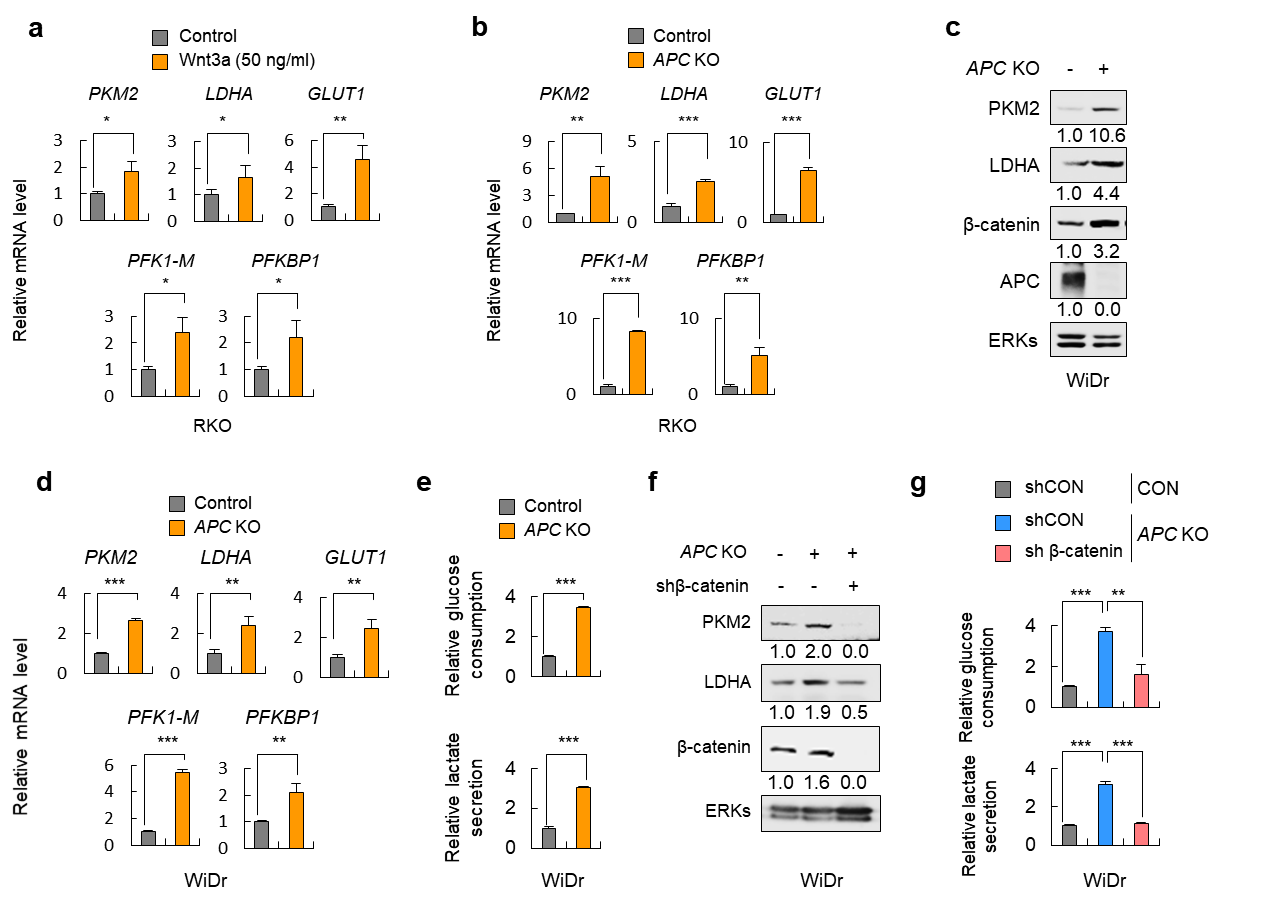
**

**Fig. S4. Effects of Wnt/β-catenin signaling activation on the expression of glycolytic genes and the Warburg effects in CRC cells. a-b** RKO cells treated with recombinant Wnt3a proteins for 24 h (**a**) and control or *APC* KO-RKO cells (**b**) were subjected to real-time PCR analyses (*n* = 3). **c-e** Control or *APC* KO-WiDr cells were harvested 24 h after seeding. The cells were used for immunoblotting (**c**) and real-time PCR analyses (**d**) to quantify the expression of glycolysis enzymes (*n* = 3). (**e**) The media collected from the cells were used for measurement of the relative glucose consumption and lactate secretion (*n* = 3). **f-g** Control-WiDr, *APC* KO-WiDr, and *APC* KO-WiDr-β-catenin knockdown cells were used for immunoblotting (**f**) and measurement of the relative glucose consumption and lactate secretion (**g**) (*n* = 3). **P* < 0.05, ***P* < 0.01, ****P* < 0.001. All data are the mean ± SD. *P* values were determined using the unpaired student’s *t*-test.

**
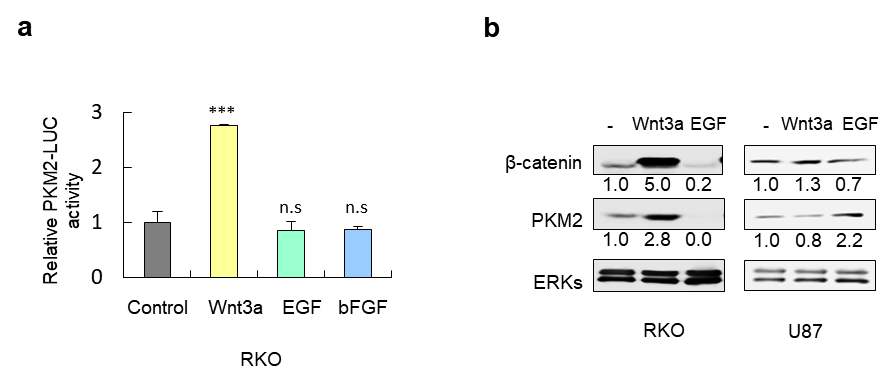
**

**Fig. S5. Effects of Wnt3a protein on the expression of *PKM2*. a** RKO cells were transfected with PKM2 promoter region-contained pGL3 luciferase reporter plasmid and pCMV–β-galactosidase vector. After 24 h, the cells were treated with 50 ng/ml of Wnt3a, EGF, or bFGF for 24 h, and the cells were subjected to the luciferase reporter assay. Data represent the mean ± s.d. (*n* = 3).**b** RKO or U87 cells were treated with 50 ng/ml of Wnt3a or EGF for 24 h, and the cells were immunoblotted with indicated antibodies. ****P* < 0.001, n.s.; not significant. All data are the mean ± SD. *P* values were determined using the unpaired student’s *t*-test.

**
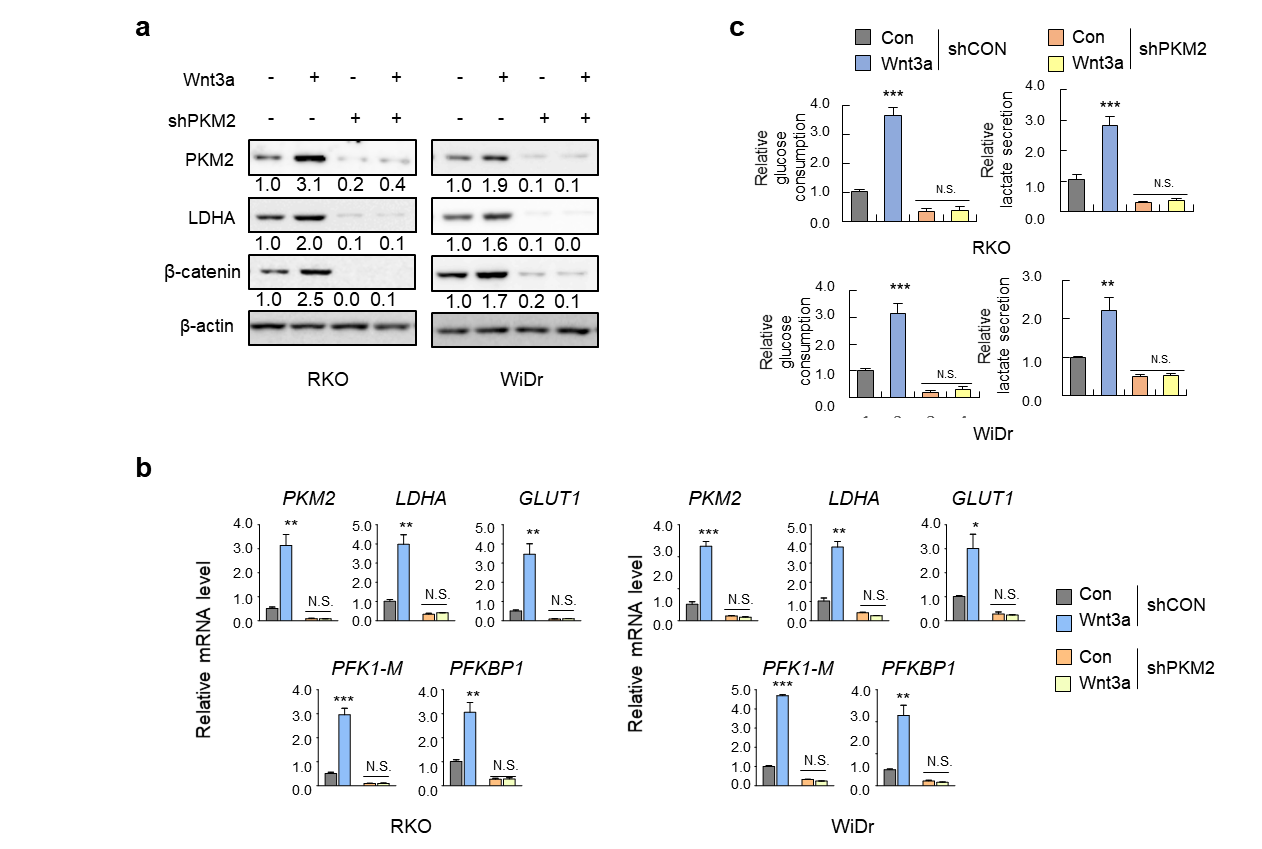
**

**Fig. S6. Effects of *PKM2* depletion on Wnt3a-induced expression of glycolytic genes and the Warburg effect in CRC cells. a-c** RKO cells and WiDr cells stably expressing the control shRNA or PKM2 shRNA were treated with 50 ng/ml of Wnt3a for 24 h, and the cells were subjected to immunoblotting (**a**), real-time PCR analyses (*n* = 3) (**b**) and measurement of the relative glucose consumption and lactate secretion (*n* = 3) (**c**). **P* < 0.05, ***P* < 0.01, ****P* < 0.001, n.s; not significant. All data are the mean ± SD. *P* values were determined using unpaired student’s *t*-test.


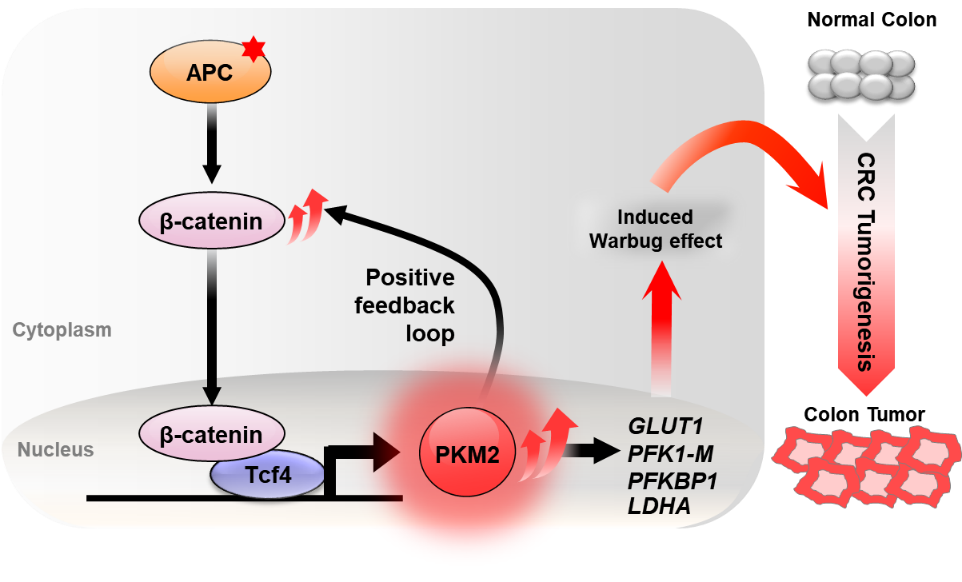


**Fig. S7. A schematic model illustrating the regulation of the Warburg effect in CRC tumorigenesis by the *APC* loss-induced β-catenin/Tcf4-PKM2 axis.** *APC* mutational loss enhances the β-catenin/Tcf4 binding on *PKM2* promoter regions and induces its transcription. The increased PKM2 upregulates the expression of glycolytic genes such as *GLUT1*, *PFK1-M*, *PFKBP1*, and *LDHA* and induces the Warburg effect, which subsequently promotes tumorigenesis. Thus, *APC* loss-induced *PKM2* is a major regulator of the Warburg effect and tumorigenesis in CRCs.
